# Saffron extract as an emerging novel therapeutic option in reproduction and sexual health: recent advances and future prospectives

**DOI:** 10.1097/MS9.0000000000002013

**Published:** 2024-04-04

**Authors:** Aman Goyal, Fatima Ali Raza, Samia Aziz Sulaiman, Abeer Shahzad, Syeda Ilsa Aaqil, Mahrukh Iqbal, Binish Javed, Prakriti Pokhrel

**Affiliations:** a Department of Internal Medicine, Seth GS Medical College and KEM Hospital, Mumbai; b Department of Internal Medicine, Atal Bihari Vajpayee Institute of Medical Sciences & Dr. Ram Manohar LohiaHospital, New Delhi,India; c Department of Internal Medicine, Karachi Medical and Dental College; d Department of Internal Medicine, Dow Medical College; e Department of Internal Medicine, Jinnah Sindh Medical University, Karachi, Pakistan; f Department of Internal Medicine, School of Medicine, University of Jordan, Amman, Jordan; g Department of Internal Medicine, Kathmandu Medical College and Teaching Hospital, Sinamangal, Kathmandu Nepal

**Keywords:** Erectile dysfunction, premenstrual dysphoric disorder, infertility, premenstrual syndrome, reproductive health, saffron

## Abstract

Saffron, derived from Crocus sativus, is gaining research attention for potential therapeutic applications. Its diverse clinical applications extend to cardiovascular health, diabetes management, sleep quality, psychiatric illnesses, and rheumatoid arthritis. Saffron’s positive effects on blood pressure, glucose levels, cognitive function, and inflammatory markers contribute to its versatility. Additionally, carotenoids like crocin and crocetin suggest anti-cancer potential. In terms of reproductive health, saffron’s impact on male reproductive health shows conflicting findings on semen parameters. However, in female reproductive health, saffron appears promising for managing dysmenorrhoea, reducing menstrual pain, regulating hormonal fluctuations, and improving overall menstrual health. Safety considerations highlight the importance of adhering to specified dosages, as excessive intake may lead to toxicity. Yet, within the therapeutic range, saffron is considered safe, relieving symptoms without serious side effects, according to clinical research. Future trials in 2023 will explore saffron’s potential in cancer therapy, diabetes management, mental health, stress response, cardiovascular health, postmenopausal women’s well-being, and chronic obstructive pulmonary disease (COPD). This ongoing research underscores saffron’s adaptability and promise as a natural treatment across various medical applications, emphasizing its efficacy. The current review, therefore, aims to provide up-to-date insights on saffron’s role particularly in the realm of reproductive health, contributing to a growing body of evidence supporting its diverse therapeutic benefits.

## Introduction

Highlights

- Ongoing research identifies saffron as promising in diverse health applications.
- Saffron’s bioactive compounds show promise in addressing conditions like erectile dysfunction, dysmenorrhoea, and enhancing libido.
- Saffron expands its role in preventive medicine, particularly in cardiovascular health.
- Caution is essential for saffron’s safe dosage, especially among specific groups such as pregnant women.

Crocus sativus L., a flower belonging to the Iridaceae family, was initially grown in the Eastern and Middle Eastern regions. Its cultivation then expanded to Mediterranean countries such as Greece. Its distinctiveness lies in the collection and drying of three red stigmas to produce saffron^1^. Initially valued for its medicinal properties, saffron is experiencing a resurgence and finding widespread use as both a spice and a colouring agent. This renewed interest in saffron has spurred investigations into its phytochemical profile and biological and therapeutic attributes^2^. Abundant carotenoids, terpenes, and saffron contain key constituents such as crocins and crocetin (both carotenoids), originating from picrocrocin, and safranal. These compounds contribute to saffron flavour and aroma. In recent years, in-depth studies of saffron have revealed that it is a rich source of multiple active ingredients, including carotenoids, flavonoids, terpenoids, amino acids, and alkaloids. These active constituents exhibit a wide array of pharmacological effects, such as anxiolytic, anti-inflammatory, anti-oxidant, antiviral, antitumor, hypoglycaemia, hypolipidemic, and memory-enhancing properties^3–6^. Crocetin could also cross the blood-brain barrier and is therefore able to reach the central nervous system, which makes it effective in the context of protecting brain tissue through anti-oxidant abilities and helps in managing neurodegenerative disease^2^.

Due to the increasing prevalence of reproductive issues in males and females, such as erectile dysfunction (ED) and dysmenorrhoea, respectively, and a rising interest in potential, natural therapeutic avenues^7^, our narrative review aims to focus on elucidating the benefits of saffron on sexual health, which could potentially aid in effectively managing and improving reproductive health issues

## Methodology

**Figure 1:**
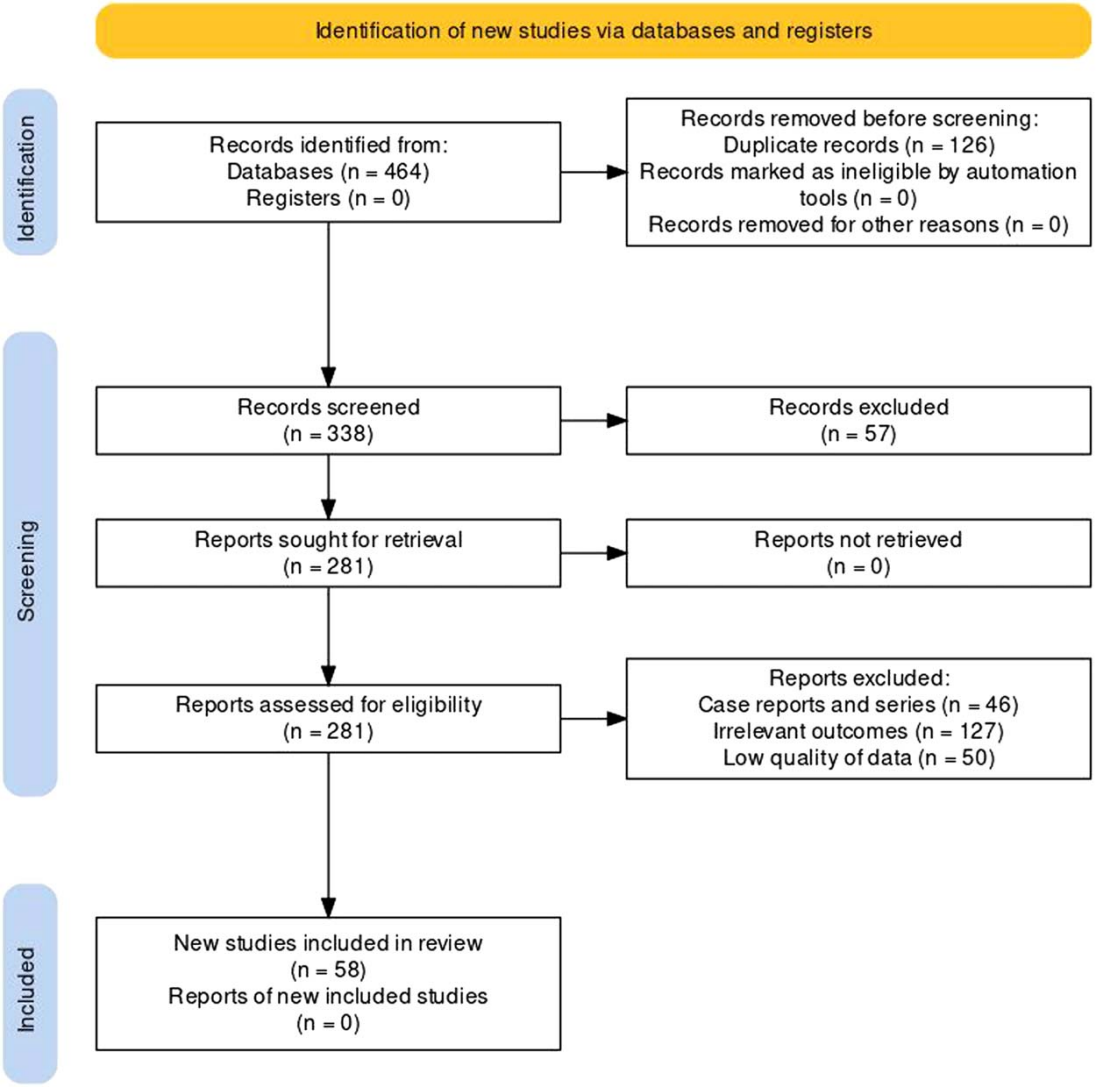
Preferred Reporting Items for Systematic Reviews and Meta-analyses (PRISMA) flow diagram for identification of studies included in the review.

We searched the PubMed/MEDLINE, EMBASE, and Scopus databases from inception to November 2023 (Fig. 1). We used keywords such as saffron, crocus, and Crocus sativus to construct the search. Mesh terms included ‘saffron,’ ‘crocus sativus,’ ‘crocus,’ ‘infertility,’ ‘premenstrual syndrome,’ ‘dysmenorrhoea,’ ‘reproductive health,’ ‘diabetes,’ ‘sleep,’ ‘COVID-19,’ ‘erectile dysfunction,’ ‘sexual function,’ ‘malignancy,’ and ‘hypertension.’ Additionally, we employed the boolean operators ‘AND’ and ‘OR’ to combine these Mesh terms.

All randomized controlled trials (RCTs), observational studies, and review articles investigating the relationship between saffron therapy and various health conditions, such as infertility, premenstrual syndrome, dysmenorrhoea, reproductive health, diabetes, sleep, COVID-19, erectile dysfunction, sexual function, and hypertension, were included in the study. Case series and reports were excluded from the study. Covidence was used for the reviewing process. Two reviewers independently screened the titles and abstracts of the retrieved studies to determine their relevance. Full texts of potentially relevant studies were obtained and further assessed for eligibility. Data were extracted into a pre-piloted Google Sheet from the eligible studies, including study design, sample size, participant characteristics, intervention used (saffron), and the results.

## The role of saffron in male reproductive health

### Erectile dysfunction

Erectile dysfunction (ED) refers to the ongoing or persistent inability to attain or maintain an erection that is satisfactory for engaging in sexual activity over a duration of at least six months^7^. ED are clinically classified into three groups: psychogenic, organic, and mixed. Psychogenic erectile dysfunction refers to the inability to establish or maintain a satisfactory erection due to psychological or relational reasons. In contrast, organic erectile dysfunction can be categorized into several types, including neurogenic, hormonal, arterial, cavernosal (veno-occlusive), and drug-induced. The vast majority of cases of organic erectile dysfunction will eventually become ‘mixed’^8^.

Because diabetes is a significant risk factor for erectile dysfunction, studies have reported that the occurrence of erectile dysfunction among diabetic men ranges from 35 to 90%^9^.

An increasing proportion of individuals across various communities worldwide are embracing plant-derived herbal remedies and medications for several reasons. These include their discontent with conventional treatments, previous positive experiences, favourable attributes linked to herbal medicine, and adherence to family traditions^10^.

Saffron, a stemless perennial herb from the Iridaceae family, is a widely used food spice known for its flavourful and aromatic properties^11^. For centuries among herbal experts, saffron has been known to have aphrodisiac-like properties. In a study from 2009 of 20 patients with erectile dysfunction, the intake of saffron for only ten days resulted in a beneficial impact on sexual function, as demonstrated by an increase in the frequency and duration of erectile events^12^. This was evaluated using the nocturnal penile tumescence (NPT) test and the International Index of Erectile Function Questionnaire (IIEF-15) at the start and end of treatment^12^. If a man with complaints of erectile dysfunction (ED) has a normal nocturnal penile tumescence and rigidity (NPTR) recording, it likely indicates psychological ED (pED), whereas an abnormal recording may indicate organic ED^13,14^. Although the aphrodisiac-like properties of saffron have been suggested for ED, the validity and efficacy remain an area of investigation; a suggested mechanism of saffron’s effects on ED is its anti-depressant effect, as depression and ED are significantly associated^13^. Furthermore, most studies do not indicate saffron’s impact on the drugs used in the treatment of ED, which would be beneficial to explore in future studies^12–14^.

Another study concluded that for patients with concomitant lower urinary tract symptoms (LUTS) and erectile dysfunction (ED), once-daily treatment with a combination of Serenoa repens (saw palmetto berries), Crocus sativus, and Pinus massoniana Bark Extract) for three months led to significant improvements (*P*<0.001) in sexual function, urinary symptoms, and quality of life. This effect was particularly pronounced in the 40-60 age group^15^.

In a 4-week, double-blind, randomized, placebo-controlled study, 36 married male patients with major depressive disorder, whose depressive symptoms had stabilized with fluoxetine but reported subjective sexual impairment, were enroled. They were randomly assigned to receive either saffron (15 mg twice daily) or placebo. The International Index of Erectile Function scale was used to assess sexual function at baseline and weeks 2 and 4. At week 4-week mark, the saffron group demonstrated remarkable improvements in the erectile function domain (*P*<0.001), intercourse satisfaction domain (*P*=0.001), and total scores (*P*<0.001) compared with the placebo group. While saffron did not significantly differ from the placebo in orgasmic function (*P*=0.095), overall satisfaction (*P*=0.334), and sexual desire (*P*=0.517) domain scores, it is worth noting that nine patients (60%) in the saffron group and one patient (7%) in the placebo group achieved normal erectile function (score>25 on the erectile function domain) by the end of the study (*p* value of Fisher’s exact test=0.005). Additionally, the frequency of side effects was similar between the two groups. This research supports the potential of saffron as a tolerable and effective treatment for fluoxetine-related erectile dysfunction^7^.

Moreover, a 4-week randomized double-blind trial was conducted, in which 36 married male patients were enroled who had complaints of major depressive disorder with stabilized depressive symptoms while taking fluoxetine but experienced subjective complaints of sexual impairment. After 4 weeks, nine patients (60%) in the saffron group and one patient (7%) in the placebo group achieved normal erectile function^7^.

A recent meta-analysis concluded that saffron was effective in men with erectile dysfunction. However, the findings revealed conflicting effects of oral saffron on semen analysis among men experiencing infertility^16^. Further research is needed to clarify the mechanism of action through which saffron influences ED.

### Male infertility

Infertility, as defined by the International Glossary on Infertility and Fertility Care (2017), is a medical condition where a clinical pregnancy is not achieved after 12 months of consistent, unprotected sexual intercourse. It can also refer to a person’s inability to reproduce, either alone or with a partner^17^. Recent studies conducted since 2017 have explored the effect of reactive oxygen species (ROS) on sperm, demonstrating that increased ROS levels are associated with diminished sperm motility, increased DNA damage, and potential germ cell apoptosis^18^. Consequently, various investigations have focused on the use of anti-oxidant agents to mitigate the impact of ROS on sperm metabolism, motility, morphology, and fertilizing capacity.

The anti-oxidant properties of saffron and its constituents have been demonstrated in various oxidative stress models. These properties may be attributed to their effects on sperm motility and infertility^16^.

In an extensive assessment of the effects of saffron on semen parameters, two distinct trials were conducted^19^. The first trial, led by Safarinejad and colleagues in rats, employed a paired *t*-test methodology and revealed significant improvements in various semen parameters, such as an increase in normal sperm morphology from 26.50±6.44% to 33.90±10.45% (*P*<0.001) and significant enhancements in sperm motility. However, it did not show a significant increase in sperm count (*P*=0.30)^20^. In contrast, the second trial conducted by Safarinejad and colleagues did not yield beneficial impacts from saffron administration, with no significant improvements in any semen parameters (*P*=0.1)^21^.

In summary, the current findings do not provide a consistent conclusion on the effect of saffron on semen parameters, necessitating further investigations with rigorous statistical analysis to draw definitive conclusions.

### The role of saffron in female reproductive health

A study conducted by Kashani *et al.* ^21^ in Iran investigated the effects of saffron on female sexual dysfunction. This research involved married women aged 18–55 years who experienced severe sexual dysfunction. The participants were administered 15 mg Crocus sativus capsules twice daily or a placebo over a period of 6 weeks. The main measure evaluated was the enhancement in the overall score of the Female Sexual Function Index (FSFI) questionnaire. The saffron group had a substantial elevation in the FSFI score in comparison to the placebo group, particularly in the areas of desire, lubrication, and contentment. These findings by Kashani *et al.* ^21^ suggest that saffron may represent a safe and effective option for ameliorating female sexual dysfunction, emphasizing the need for further robust studies in this domain.

### Dysmenorrhoea

Dysmenorrhoea, or painful menstrual cramps, is a common gynaecologic complaint that affects 50–90% of women worldwide^22^. It is defined as pain during menses in the absence of an identifiable pathological lesion. However, pain is believed to be related to the production of prostaglandins, which induce myometrial contractions and contribute to uterine ischaemia and sensitization of afferent nerve fibres to painful stimuli^23^. Dysmenorrhoea can be classified as either primary or secondary dysmenorrhoea. Primary dysmenorrhoea is a lower abdominal pain that occurs during the menstrual cycle and is not associated with other diseases or pathologies^24^. In contrast, secondary dysmenorrhoea is usually associated with pathologies inside or outside the uterus^25^. Dysmenorrhoea is a common complaint among women during their reproductive age. Dysmenorrhoea is associated with significant emotional, psychological, and functional health impacts^26^. Most treatments for dysmenorrhoea use knowledge of PG production and act through the disruption of specific steps in PG formation^23^. While nonsteroidal anti-inflammatories and oral contraceptive pills are commonly used in the treatment of dysmenorrhoea,^27^, researchers are exploring alternative therapies that may offer similar benefits naturally, including the use of saffron^28^. Studies have shown that saffron may be beneficial in treating dysmenorrhoea as it has been found to efficiently relieve menstrual pain. In a pilot RCT, saffron was combined with celery seed and anise extracts and was found to be effective in reducing the severity and duration of menstrual pain. However, since the herbal drug contains three herbs, this effect cannot be attributed specifically to saffron^23^.

Additionally, saffron has been found to have positive physiological and psychological effects in managing dysmenorrhoea, premenstrual syndrome (PMS), and irregular menstruation. Since dysmenorrhoea is often associated with elevated cortisol levels and hormonal imbalance, a study found that exposure to saffron odour led to a decrease in cortisol levels observed after saffron exposure, which may help alleviate the symptoms of dysmenorrhoea. Additionally, the increase in estradiol, a form of oestrogen, suggests that saffron could potentially regulate hormonal fluctuations during the menstrual cycle^16^.

Although more research is needed to fully understand the benefits of saffron in the treatment of dysmenorrhoea, these findings offer promising insights into potential alternative therapies for this common and often debilitating condition.

### Premenstrual syndrome and premenstrual dysphoric disorder

PMS is one of the most common problems that deteriorates the quality of life of women of reproductive age. PMS refers to the mental, physical, and behavioural changes, such as sadness or hopelessness, anxiety or tension, extreme moodiness, marked irritability, or anger that occur during the luteal phase of the menstrual cycle, that is, after ovulation and before the onset of menstruation^29,30^.

A study conducted by Granda *et al.* ^30^ suggests that around 30% of women suffer from moderate to severe symptoms of PMS with at least one of the four major symptoms (anger/irritability, anxiety, tearfulness/emotional lability, and depressed mood), and approximately 80% of women have mild PMS symptoms such as pain, fatigue, irritability, decreased home and work activities, etc^31^. Premenstrual dysphoric disorder (PMDD), the most severe form of PMS, affects ~3–5% of women of childbearing age^31^. The prevalence of PMS is high in low-middle-income regions, and most of these women do not take any treatment to reduce PMS symptoms, probably due to deterioration in law and order with people living under long-term curfew orders, as suggested by Shershah *et al.* ^32^.

The effectiveness of saffron in the treatment of gynaecological problems has been studied in detail. For instance, saffron improves the symptoms of PMS and PMDD. Saffron has various modes of action, including anti-inflammatory, anti-nociceptive, anticonvulsant, and anti-depressant^16^. The effect of saffron on PMS symptoms comes from its ability to influence the neurotransmitter serotonin.

Fluctuations in steroid hormone levels, including testosterone and estradiol, due to increased cortisol levels during the follicular phase play a crucial role in the development of PMS symptoms^19^. A double-blind placebo-controlled trial studied the effectiveness of saffron odour in 35 female college students with regular menstrual cycles and PMS symptoms. Saffron significantly improved PMS symptoms by decreasing cortisol levels after short-term exposure^16^.

Another double-blind randomized clinical trial aimed at investigating the effects of saffron on PMS and PMDD reported a 50% reduction in the severity of PMS symptoms evaluated using a standardized questionnaire in approximately 70% of women of reproductive age in the saffron group (30 mg). A few tolerable adverse effects, mainly headache and low appetite, have also been reported^33^.

A recent trial conducted in 2020 assessed 120 females with diagnosed PMDD and proved that saffron is a successful herbal agent for the treatment of PMDD by improving symptoms of depression with minimal side effects^34^.

### The use of saffron in enhancing sexual function

Saffron supplements are widely consumed globally, as they are believed to possess aphrosisiac properties. Several studies, conducted on human subjects, have indicated the potential benefits of saffron, especially its crocin component^12,34^, in enhancing sexual function. Further research is necessary to establish and confirm the efficacy of saffron in improving sexual function.

The beneficial effects of saffron on sexual function can be attributed to its active compounds, crocetin and crocin. These compounds have been shown to induce endothelium-dependent relaxation by increasing eNOS activity^35^ and to contribute to the relaxation of the corpus cavernosum smooth muscle via inhibition of extracellular Ca2+ influx and intracellular Ca2+ release^36^. These mechanisms contribute to the relaxation of smooth muscle cells in the penis, leading to increased blood flow and ultimately, erection.

According to Shamsa *et al.* ^12^, taking 200 mg saffron can enhance sexual performance, pleasure, orgasm, desire, and overall satisfaction. In a different study, Kashani and colleagues found that using 30 mg of saffron for one week can effectively improve certain sexual issues induced by fluoxetine, a drug used to treat depression, panic attacks, obsessive-compulsive disorder, bulimia, and premenstrual dysmorphic disorder, such as lubrication and arousal^37^. Another study reported that saffron administration improved erectile function, sexual satisfaction, orgasm, sexual desire, and overall satisfaction among men^12^.

These findings suggest that saffron may have a significant impact on libido and quality of life in individuals struggling with sexual dysfunction.

### Constituents of saffron

The constituents of saffron give the characteristics and potential benefits, as demonstrated by Table 1.

**Table 1:** The roles, characteristics, and potential benefits of the constituents of saffron.

Constituent	Role	Characteristics	Potential benefits	References
Crocin	Carotenoid compound	Converted to crocetin Provide colour, sweet taste, anti-oxidant properties	Potent anti-oxidant and anti-inflammatory properties	^36–38^
Crocetin	Carotenoid compound	formed by hydrolysis of crocin, Provide colour, sweet taste, anti-oxidant properties	Potent anti-oxidant and anti-inflammatory properties	^38^
Safranal	Aromatic compound	Formed from picrocrocin, provides aroma and flavour	Anti-oxidant, anti‐inflammatory 2, and anti-anxiety activity, anti-depressant, neuro protection	^37,38^
Picrocrocin	Bitter compound	Converts to safranal, Contributes to bitterness	Anti-oxidant and anti-inflammatory properties	^38^
Zeaxanthin	Carotenoid compound	Picrocrocin is the degradation product of Zeaxanthin	anti-oxidant and anti-inflammatory properties	^37,38^

### Methods of preparation for saffron

Different forms of saffron are found and made for consumption, although some could be used as drug abuse, as shown in Table 2.

**Table 2:** Preparation forms and usage of saffron.

Preparation Form	Different usage in literature	References
Oral tablets/capsules	Depression and mood disorders Erectile dysfunction, Hypertension	^7,16^
Topical gel	Erectile dysfunction in diabetics	^38^
Saffron syrup	Fatigue reduction in patients with multiple sclerosis, Facilitation of labour in combination with honey syrup	^36–40^
Saffron spice and colour	Food seasoning, colouring agent Faviring agent in food And cosmetics	^2,11^

### Safety of saffron use

Research on the toxicological effects of saffron has shown that it has a safe dosage of 1.5 g/day based on lethal dose value (LD50) values, while toxic dosages of 5 g/kg and 20 g/kg can cause death. Ingesting more than 10 g/day can lead to various side effects, including haematuria, diarrhoea, headache, hypomania, dizziness, nausea, vertigo, and gastrointestinal bleeding, while more severe toxicity can cause numbness, tingling in the hands and feet, and yellowing of the skin and eyes because of the precipitation of yellow pigments on the skin and conjunctiva. Pregnant women should take saffron with caution because of the potential embryonic miscautions and increased miscarriage rates^36^.

Saffron has been found to reduce platelet counts and elevate creatinine (Cr) and blood urea nitrogen (BUN) levels at a dose of 200 mg/day, although no significant difference has been observed in many studies^36–39^.

Research on the toxicological effects of saffron has shown that it has a safe dosage of 1.5 g/day based on lethal dose value (LD50) values, while a toxic dosage is considered to be 5 g/kg and a dosage of 20 g/kg can lead to death. Ingesting more than 10 g per day can lead to various side effects, including haematuria, diarrhoea, headache, hypomania, dizziness, nausea, vertigo, and gastrointestinal bleeding, while more severe toxicity can cause numbness, tingling in the hands and feet, and yellowing of the skin and eyes due to the precipitation of yellow pigments on the skin and conjunctiva. Saffron possesses a wide therapeutic index and is generally safe for use as demonstrated in clinical trials. Although it is acknowledged that saffron can be toxic at very high doses, with doses exceeding 5 g considered potentially harmful and 20 g/day possibly lethal, the common effective doses used in clinical trials typically range from 30 to 50 mg/day. This substantial difference between the effective clinical doses and potentially toxic levels underscores the safety of saffron within the therapeutic range. Therefore, the wide therapeutic index of saffron suggests that it can be safely administered^39^.

Numerous trials have been conducted to assess the safety of saffron, revealing important insights into its potential toxicity and side effects that must be considered to ensure its impact on health. In a 2021 double-blinded study involving 86 perimenopausal women, the effects of a 14 mg dose of saffron extract (affron) administered twice daily were examined. This study revealed significant improvements in mood and psychological symptoms, particularly a substantial reduction in anxiety and depression scores. This suggests that the 14 mg dose of saffron showed promise in addressing psychological symptoms during perimenopause while being well tolerated with no major adverse events reported^40^. In a 2022 clinical trial on Crocus sativus (saffron) for treating female sexual dysfunction, married women aged 18–55 years with severe sexual issues were divided into two groups. One group received 15 mg Crocus sativus capsules twice daily, while the other received placebo for 6 weeks. Both groups showed improvements in their total scores, but the saffron group exhibited a significant advantage (*P*=0.050). Notably, by the 6th week, the saffron group showed a remarkable 62% improvement from baseline, with particular benefits in desire, lubrication, and satisfaction within the female sexual function index, with no reported side effects^40^. A meta-research study conducted until 3 April 2021, aimed to assess the clinical evidence from meta-analyses regarding the efficacy and safety of saffron (Crocus sativus L.). The study identified 19 systematic reviews and meta-analyses published between 2013 and 2021. The results revealed that saffron significantly improved various health parameters, including fasting blood glucose, waist circumference, diastolic blood pressure, total cholesterol, low-density lipoprotein cholesterol, and symptoms of depression, cognitive function, and sexual dysfunction when compared to control groups, primarily placebos. Common side effects included nausea, dry mouth, poor appetite, and headaches, and no serious adverse reactions were reported. Available evidence suggests that saffron is safe for medicinal use and can improve various clinical outcomes^41^.

Overall, saffron appears to be safe when used within common therapeutic doses, but higher doses can lead to toxicity; therefore, careful consideration of dosage is essential.

### Application of saffron beyond reproductive health

Saffron has many clinical applications apart from its use in sexual dysfunction. Many clinical trials have been conducted that have proven the efficacy of saffron as an analgesic and in the treatment of cardiovascular disease, cancer, immune disorders, insomnia, psychiatric illnesses, RA, inflammatory disorders, and diabetes^38^.

### Cardiovascular benefits

Saffron has been recognized to have the potential to mitigate cardiovascular risk factors through its anti-atherosclerotic, anti-oxidant, antidiabetic, hypotensive,anti-ischaemic, anti-platelet aggregation and hypolipidemic effects; this shows her promise as a treatment option^42^. In a RCT, the results demonstrated that saffron at a dose of 400 mg significantly decreased standing systolic blood pressure and mean arterial pressure and increased serum sodium, BUN, and Creatinine (Cr)^42^. Although some studies showed no significant effect of saffron on systolic blood pressure, it seemed to reduce diastolic pressure^42^.

### Diabetes mellitus

Clinical research and experimental models have provided evidence that saffron extracts and its bioactive components have the ability to alleviate diabetes. In a 2019 double-blind RCT, researchers examined the impact of saffron extract on different health indicators in a group of 64 individuals with type 2 diabetes who were previously using oral antidiabetic medications. Subjects were randomized at random to receive either 15 mg saffron or placebo capsules on a daily basis for a duration of 3 months. Following the 3-month intervention, the saffron group demonstrated noteworthy decreases in fasting plasma glucose (FPG), cholesterol, LDL cholesterol, and LDL/HDL ratio in comparison to the placebo group. Nevertheless, there were no substantial disparities observed in glycated haemoglobin (HbA1c), HDL cholesterol, atherogenic index (API), and TG levels between the two groups^43^. In a 2019 comprehensive review, researchers studied the impact of saffron supplementation on glucose levels and lipid profiles in patients with diabetes. The review encompassed six research, and the findings demonstrated a noteworthy decrease in the serum concentrations of total cholesterol (TC) and TG following saffron administration. Furthermore, there was a notable elevation in levels of high-density lipoprotein (HDL). These findings indicate that using saffron supplements may improve the lipid profile, namely by lowering TC and TG levels while boosting high-density lipoprotein (HDL) levels^43^.

### Sleep quality

Saffron extract may have a positive impact on sleep quality. A RCT published in 2021 found that after 6 weeks of saffron supplementation, participants experienced increased time in bed, improved ease of getting to sleep, and enhanced sleep quality, latency, duration, and global scores compared to those who took a placebo. This is promising for individuals with mild-to-moderate sleep disorders associated with anxiety^44^. Additionally, a systematic review conducted up to 2022 identified five randomized clinical trials with 379 participants from three countries to evaluate the effects of saffron on sleep quality. The trials included placebo comparisons, and the results suggested that saffron has a beneficial influence on the duration and quality of sleep. Saffron, specifically its constituents crocin and safranal, appears to induce hypnotic effects by increasing sleep duration. The research conducted thus far provides initial support for the safe use of saffron as a potential natural remedy to improve sleep quality^45^.

### Psychiatric benefits

Saffron affects various psychiatric illnesses, notably Depression, Dementia and Alzheimer’s disease. In an umbrella meta-analysis conducted in June 2021, saffron (Crocus sativus L.) was examined for its potential impact on depression. The analysis incorporated findings from seven meta-analyses exploring the effects of saffron on depression symptoms. The study revealed that saffron intake, as of 2021, was associated with a significant reduction in BDI (Beck Depression Inventory) scores, with an effect size of −3.87 (95% CI: −5.27, −2.46). This indicates that saffron had a positive influence on reducing depressive symptoms, as measured by the BDI. This suggests that saffron intake may contribute to the alleviation of depression, as indicated by the reduced BDI scores. It is likely to be more effective when used in conjunction with other therapeutic interventions or treatment strategies^46^. In another meta-analysis conducted in January 2019, the efficacy of saffron in the treatment of mild-to-moderate depression was evaluated. The meta-analysis, conducted following PRISMA guidelines, involved a review of RCTs. These trials were designed to assess the efficacy of saffron in patients with mild-to-moderate depression. The study included data from patients using pharmacological doses of saffron per os (by mouth) and compared saffron with placebo or active controls. The analysis revealed that saffron had a significant effect on depression severity. The results indicated that saffron was notably more effective than the placebo (g=0.891; 95% CI: 0.369–1.412, *P*=0.001). Furthermore, saffron demonstrated non-inferiority when compared with the tested anti-depressant drugs (g=−0.246; 95% CI: −0.495 to 0.004, *P*=0.053). This meta-analysis provides strong evidence that saffron is an effective treatment option for mild-to-moderate depression. It not only outperformed a placebo but also showed similar efficacy to commonly prescribed anti-depressant medications. These findings highlight saffron as a promising natural alternative for the management of depressive symptoms^47^. However, in 2020, the focus was on the effectiveness and safety of saffron in managing mild cognitive impairment and dementia. Saffron, a traditional herbal remedy with a history of application to various health conditions, has recently been explored for its potential in combating dementia. The study included a search of major English and Chinese databases to identify relevant RCTs up to May 2019. The analysis, based on four RCTs, revealed significant positive outcomes. Saffron was found to significantly enhance cognitive function, as measured by the Alzheimer’s Disease Assessment Scale-cognitive subscale and Clinical Dementia Rating Scale-Sums of Boxes (CDR-SB), in comparison to placebo^48^.

### In rheumatoid arthritis

Saffron exhibits promising therapeutic effects in the management of RA. A randomized, double-blind, placebo-controlled experiment was conducted to assess the impact of saffron supplementation on clinical outcomes in patients with active RA. The results of the trial demonstrated that saffron supplementation yielded favourable benefits in a range of clinical outcomes. Following a 12-week period of using saffron supplements at a daily dosage of 100 mg, notable enhancements in many parameters associated with RA were noted. The enhancements consisted of a reduction in the quantity of painful and swollen joints, diminished pain intensity as assessed by the visual analogue scale, and a lower disease activity score. These parameters are commonly used for assessing the severity of RA^49^. The saffron intervention led to significant improvements in both the Physician Global Assessment and erythrocyte sedimentation rate. In addition, the saffron group exhibited a reduction in high-sensitivity C-reactive protein levels. While there were changes in certain inflammatory markers and signs of oxidative stress, the saffron and placebo groups did not show statistically significant differences. According to this study, adding saffron to the treatment regimen of patients with RA has a beneficial and noteworthy effect on their clinical outcomes^50^.

### Anti-inflammatory and analgesic properties

Therefore, the anti-inflammatory properties of saffrons cannot be overlooked. In a clinical trial conducted in 2023, the role of crocin in reducing serum inflammatory markers (IL-6 and TNF-α) in chronic obstructive pulmonary disease (COPD) was investigated. Saffron, similar to crocin, contains compounds with anti-inflammatory properties. Studies have suggested that saffron can help to reduce inflammation, making it a potential natural remedy for managing various inflammatory conditions. The active components of saffron, such as crocetin and crocin, are believed to contribute to its anti-inflammatory effects, which can benefit individuals with conditions characterized by inflammation, although specific studies on the role of saffron in inflammation are not covered in this particular research^51,52^. From this study, it is evident that dietary polyphenols, including Crocus sativus L. extract (saffron), are being studied for the treatment of rheumatoid arthritis (RA). A meta-analysis of 47 RCTs involving various dietary polyphenols has suggested that these compounds may have a positive impact on RA treatment. Dietary polyphenols, such as saffron, appear to improve disease activity scores for 28 joints, reduce inflammation levels (e.g. C-reactive protein or CRP), lower erythrocyte sedimentation rate (ESR), and enhance oxidative stress parameters^52^.

Saffron has also demonstrated anti-analgesic properties^53^. In a clinical trial conducted in patients undergoing methadone maintenance treatment (MMT) programs, the effect of crocin, the main active constituent of saffron, was evaluated. This study aimed to assess the effect of crocin administration on withdrawal syndrome, craving, and cognitive function in these patients. Patients were divided into two groups: the intervention group, which received 30 mg/day of crocin, and placebo group. The 12-week intervention showed that crocin resulted in a significant improvement in craving and withdrawal symptom scores compared to the placebo group. However, it did not have a significant effect on cognitive function parameters, including TMT, FAS test, and DGSP score. This suggests that crocin, found in saffron, has the potential to alleviate withdrawal symptoms and cravings in opioid patients under MMT programs without affecting their cognitive functions^53^.

### Anti-malignant properties

Research provides strong support for the use of saffron, particularly its carotenoids, crocin and crocetin, in cancer chemotherapy and chemoprevention. In this study, it was highlighted that saffron carotenoids have been extensively studied for their various biological activities, including their role in inhibiting tumour growth and inducing cancer cell death. This study collected information from numerous scientific databases and identified a substantial body of evidence. When searching for a relationship between saffron and cancer, approximately 150 articles were found. Similarly, there were approximately 60 articles related to crocin and cancer, and ~55 articles related to crocetin and cancer. Additionally, over 16,000 reports have been found when searching for carotenoids and cancer. Collectively, research findings highlight that saffron and its carotenoids exhibit chemopreventive activities, including anti-oxidant effects, cancer cell apoptosis, inhibition of cell proliferation, enhancement of cell differentiation, and modulation of cell cycle progression. These findings emphasize the potential of saffron compounds as valuable components for cancer therapy and prevention^54^.

### In COVID-19

Saffron has also demonstrated a role in mitigating the risk of complications arising from COVID-19 infection, which could occur due to increased inflammation and cytokine storms, concludingly resulting in multi-organ failure and a risk of mortality, as demonstrated by Hegazy *et al.* ^55.^ Compounds within saffron, such as crocetin esters, picrocrocin, and safranal, possess strong anti-oxidant and anti-inflammatory properties, making them promising for managing COVID-19 and its respiratory symptoms. Crocin, a component of saffron, reduces the COVID-19-related cytokine cascade and downregulates ACE2. Additionally, studies have suggested that saffron astragalin and crocin might inhibit key components of the SARS-CoV-2 virus. Although saffron shows promise as a preventive and adjunct treatment for COVID-19, further research, particularly through randomized clinical trials using inflammatory biomarkers, is needed to establish its clinical efficacy, especially in resource-poor settings where access to pharmaceutical drugs is limited^56^. Furthermore, Rathore *et al.* ^57^ have found that whether saffron can act as an adjunct treatment along with standard of care management for frequently encountered complications with high risk of morbidity and mortality in patients with COVID-19, such as upper gastrointestinal bleeding, heart failure, and respiratory failure is unclear.

### Limitations

The current review is not one lacking limitations that could be improved on to provide a more nuanced understanding of the role saffron has on reproductive health. First, since the review is based on other studies, the heterogeneity amongst the existing literature regarding study design, including variations in dosages, sample size, and outcome measures, could limit the generalizability and accuracy of the current review. Second, some studies also lack adequate randomization and blinding, which could potentially compromise the internal validity of the findings. Another limitation is that our review did not include statistical analysis of the data gathered, which may limit its generalizability and robustness. However, since most studies did not gather the results from a control group, statistical analysis could not be included as part of the current review, which warrants further prospective studies exploring saffron’s effects on reproductive health.

Lastly, the variation in the composition and quality of saffron preparations amongst studies also poses a challenge for generalizing the findings of the current review, which highlights the need for future research to evaluate the comparative efficacy of different saffron products.

### Future implications

The findings of the current review could contribute to the development of clinical guidelines and recommendations in the context of reproductive health, playing a crucial role, especially in patient populations seeking alternative or adjunctive approaches. Public health education can raise awareness of the potential benefits of saffron and provide evidence-based information on its efficacy, safety, and appropriate use in relation to reproductive health, as well as in improving overall health and preventing certain conditions.

The current review also sheds light on the importance of future trials focusing on elucidating the underlying mechanisms through which saffron exerts its effects on reproductive health. This can help validate its role in hormonal regulation, signalling, and inflammatory pathways in both animal and human subjects. Further research could also emphasize the recommended dosages and adverse effects in specific populations, such as saffron use in pregnancy. Furthermore, studies with larger sample sizes and longer-term follow-ups could highlight the efficacy and safety of saffron in diverse populations with different histories of reproductive health conditions. Further research evaluating the efficacy of saffron as an adjunct to novel, effective medications such as bempedoic acid and inclisiran to reduce cardiovascular morbidity and mortality should also be explored, as cardiac disease remains the number one cause of death^58^. Focusing research efforts on this area would yield the maximum return on investment and may further improve the cost-effectiveness of these novel and expensive medications.

## Conclusions

In conclusion, this study highlights the multifaceted potential of saffron as a natural remedy for addressing various health concerns. From its historical use as an aphrodisiac for modern applications in treating conditions such as erectile dysfunction, dysmenorrhoea, premenstrual syndrome, and enhancing libido, saffron’s bioactive compounds have shown promising effects. Additionally, the potential role of saffron in mitigating cardiovascular risk factors suggests a broader scope of its application in preventive medicine. However, it is important to be mindful of saffron’s safe dosage limits and potential adverse effects, especially for specific populations such as pregnant women. As research continues, saffron holds promise as a natural resource for promoting well-being and addressing a range of health concerns. Our review highlights the need for future research to highlight the efficacy and mechanisms of saffron in regard to reproductive health.

## Ethical approval

The information provided in the manuscript does not require an ethics application or approval.

## Consent

No patients were sorted. Not applicable.

## Source of funding

Not applicable.

## Author contribution

A.G.: conceptualization, supervision, resources, manuscript writing, revision. F.A.R.: conceptualization, supervision, resources, manuscript writing, revision. S.A.S.: conceptualization, supervision, resources, manuscript writing, revision. A.S.: manuscript writing and editing. S.I.A.: manuscript writing and editing. M.I.: manuscript writing and editing. B.J.: manuscript writing and editing. P.P.: manuscript writing and editing.

## Conflicts of interest disclosure

The authors declare no conflicts of interest.

## Research registration unique identifying number (UIN)

Not applicable for narrative review.

## Guarantor

Aman Goyal.

## Data availability statement

Not applicable.

## Provenance and peer review

Not applicable.
